# “We Need to Raise Awareness and Never Give Up”: Israeli Druze and Muslim Arab Kindergarten Teachers’ Proactivity When Facing the Sexual Abuse of Their Students

**DOI:** 10.3390/bs14020142

**Published:** 2024-02-16

**Authors:** Noah Bar Gosen, Laura I. Sigad, Jordan Shaibe, Amani Halaby, Efrat Lusky-Weisrose, Dafna Tener

**Affiliations:** 1 Department of Inclusive Education, Faculty of Graduate Studies, Oranim College of Education, Kiryat Tiv’on 3600600, Israel; noamikael@gmail.com (N.B.G.); laura_s@oranim.ac.il (L.I.S.); jordan.shaibe@mail.huji.ac.il (J.S.); amosh83@yahoo.com (A.H.); 2 The Paul Baerwald School of Social Work and Social Welfare, Mount Scopus Campus, Hebrew University of Jerusalem, Jerusalem 9190500, Israel; dafna.tener@mail.huji.ac.il

**Keywords:** kindergarten teachers, childhood sexual abuse (CSA), Druze communities, Muslim communities, coping

## Abstract

Kindergarten teachers are expected to lead the intervention process in cases of child sexual abuse (CSA) in their kindergarten. This study examines the proactive role of Druze and Muslim Arab kindergarten teachers in addressing and coping with the CSA of their kindergarten students in Israel. A qualitative thematic analysis was used to investigate the semi-structured interviews conducted with eight Druze Arab and six Muslim Arab kindergarten teachers. Three distinct themes were revealed. The first theme described the participants’ fear and concern for their personal children and themselves when dealing with CSA incidents involving their students. The second and third themes described their proactive coping on two fronts: (1) inside their homes to protect their own children and (2) as educators within religious communities, using professional and religious principles to support CSA survivors and raise awareness among parents. The results emphasized the personal burden on kindergarten teachers coping with CSA in their kindergarten and, as mainly expressed by Druze kindergarten teachers, the contribution of religious values to CSA intervention and prevention processes among their students and communities. Thus, there is a need for comprehensive support that considers ethnic and religious characteristics and will be available to kindergarten teachers facing CSA in their kindergarten.

## 1. Introduction

Child sexual abuse (CSA) is recognized globally as a serious social issue, defined as sexual activities involving a child under 18. These acts can be physical or non-physical, where the child cannot genuinely consent, highlighting the child’s vulnerability and the power imbalance in such situations [1]. Whilst there is extensive international literature on child sexual abuse (CSA), research has, for the most part, drawn upon survivors’ accounts and less upon professionals contending with these instances in their everyday work [2].

Kindergarten teachers are responsible for all aspects of the well-being related to their students [3,4]. This role is challenging and escalates in times of stress and extreme incidents, such as CSA. However, despite their central role in identifying help-seeking CSA-related behaviors among their students [5], the critical field of kindergarten teachers coping with these cases is in its infancy.

Moreover, community and family characteristics, such as religion and tradition, can also influence the way kindergarten teachers feel, react, and behave when facing CSA incidents in their kindergarten. In return, the effects of CSA within the kindergarten ecological system can extend beyond the classroom into the community and family spheres [6]. The present study aims to fill the gap of an overlooked phenomenon by analyzing the challenges and insights of Druze and Muslim Arab kindergarten teachers in Israel contending with CSA by an adult perpetrator, among who belong to communities characterized by conservative, traditional, and religious values.

In Israel, kindergarten teachers are the formal authority of the kindergarten. They are responsible for their students and are the kindergarten’s external representatives. As such, they are expected to maintain constant communication with their students’ parents [7,8]. There is a structured distinction between the kindergarten and primary school systems in the Israeli education system. It creates a pedagogical context in which the kindergarten teacher is the primary educational leader and caregiver, as opposed to schoolteachers who work within a professional sphere where peer teachers and management and organizational personnel share their burden. Thus, kindergarten teachers’ work conditions can result in a solitary experience [7,9], despite the availability of professional supervision [4,7]. Due to their position as educational managers and leaders, kindergarten teachers’ perceptions and experiences are vital to understanding the challenges embedded in intervention processes when facing CSA incidents among kindergarten students.

### 1.1. Socio-Cultural Context of the Study: Israel and the Druze and Muslim Arab Communities

Israel’s cultural diversity has been a topic of debate, with conflicting views regarding it being pluralistic and multicultural versus deeply divided and conflictual [10]. The population of Israel consists of various ethnic and religious groups [11], with Jews comprising the majority (approximately 75%) and Arabs comprising a significant minority (approximately 20.7%) [12]. The Arab minority in Israel is highly diverse, comprising various socio-cultural groups, including Muslim, Christian, and Druze [13]. The various Arab societies in Israel have integrated aspects of both Western and individualistic secular Jewish cultures, while maintaining their own traditional collectivist norms [13]. Studies have found that the overall Arab society typically views child abuse from the perspective of hierarchy, commitment to the extended family, and social cohesion [14]. Enforcing the law against sexual predators may harm the family honor and reputation. Consequently, families may turn against the victim in the name of family cohesion, rather than seeking punishment or revenge [6].

The Muslim Arab community in Israel claims to hold values of interdependence, collectivism, hierarchy, family honor, and social cohesion in high regard [15]. However, there is an overall distrust of Israeli authorities as a result of ongoing cultural conflicts and a sense of betrayal in encounters with government officials. Such distrust may lead to an underreporting of child abuse [14]. On the other hand, the Druze population in Israel makes up 7.6% of the country’s Arab population [16]. Although part of the Arab population in Israel, the Druze community has unique characteristics that differ from the other Arab subgroups [17]. While the Druze community maintains its own traditions and values, it has also adopted Israeli norms [18]. For example, Druze community members often serve in the Israeli military [19], whereas Muslim community members do not.

A community’s political, organizational, and geographical contexts are also vital to consider to better understand approaches to CSA [20]. In Israel, less funding and fewer resources are provided for Arab communities, including schools and services related to child protection and education as well as for positions of social welfare professionals, than for Jewish communities [21]. Consequently, this lack of services such as funding, facilities, resources, and extra-curricular activities may significantly impede the community’s access to CSA prevention and intervention [21]. Consequently, such inequality can diminish the resources available for necessary CSA services.

### 1.2. Sexual Abuse of Children in Israel

The suspicion of CSA in Israel constitutes approximately 11% of educational professionals’ referrals to child welfare services [22]. Nearly one-quarter of referrals to the Israeli Rape Crisis Center in 2020 concerned minors [23]. Most children are reported to have been abused by perpetrators they know [22,23], with some harmed within the educational system itself [23].

Encouraging students’ healthy sexual development is one of the complicated challenges kindergarten teachers face. It can be challenging to address this subject while countering resistance from parents or community members. Another contribution to the challenge can be the ambiguous line between typical developmental curiosity and ‘abnormal’ sexual behaviors [24], increasingly so when the interaction is between two children [25]. The leading role as kindergarten managers with its solitary components, together with their extensive responsibilities and love for children, can raise scruples, doubts, and stress. Ultimately, it can make the kindergarten teachers question their professional identity as well as being a source of strain on their personal lives [26]. Consequently, their exposure to the distressful knowledge that comes with cases of CSA invades their personal lives. The toxic knowledge to which they are exposed affects their parental practices, especially those aimed to protect their own children, as well as their social interactions within their communities [27].

### 1.3. Sources of Support for Kindergarten Teachers Encountering CSA

When encountering CSA cases in their kindergarten, teachers are required to operate professionally. Therefore, they are expected to attend to the needs of the victimized students who have been sexually abused. They must also instruct the support staff on expected conduct such as extra care or attentiveness to signs of distress as well as expected reactions. In addition, the teachers need to inform the student’s parents of the CSA and its implications, as well as assist parents by offering support and guidance. This entails balancing the students’ right to confidentiality and the parents’ rights and demands to know what happened. As professional educators, they are bound to confidentiality. Therefore, they cannot reveal incidents that occurred in their kindergarten or those related to any of their students to people outside the kindergarten sphere [28], unless it is to the families involved or the formal authorities. This constraint limits their ability to share their burden and be supported within their family circle.

### 1.4. Kindergarten Teachers Coping with CSA Incidents in the Muslim and Druze Arab Communities

The traditional characteristics of Arab society emphasize patriarchy, collectivism, chastity, and family honor [29,30,31]. The dependence nature embedded in the family structure in traditional societies makes the family the main source of protection for the individual. As such, people are less inclined to turn to formal authorities to seek justice [6]. Rather, they prefer to follow family and community guidelines and settle CSA incidents within the family. Kindergarten teachers need to navigate between their will to help their students, follow formal mandatory guidelines from the Ministry of Education [28], and address the parents and community’s values in a manner that will promote collaboration and ensure the safety of all the included parties [32]. Thus, Arab teachers in Israel have described a sense of existing within dual and often conflicting identities, experiencing pressure to meet the differing expectations of the Ministry of Education and Arab society [33].

There are a variety of materials that all educators, including kindergarten teachers, can access when coping with the CSA of their students. For example, the state has detailed guidelines for reporting and addressing abnormal sexual behaviors in kindergartens [28]. Furthermore, the education system’s staff portal offers online self-learning materials for identifying distress and abuse [34]. Information from various other organizations can be accessed online as well (e.g., [35]). Unfortunately, although materials are available in Arabic, they are often directly translated rather than adapted to the general Arab culture or specific Muslim and Druze religions. While there is no breakdown as to whether or not educational materials should be revised to adapt religious or cultural features, it has been found that these materials are less supportive of kindergarten teachers’ intervention processes in cases when Druze or Muslim Arab students are involved [6,36,37]. Additionally, although supervisors, counselors, and psychologists can assist kindergarten teachers in times of need, as mentioned earlier, they are frequently isolated and can only depend on themselves [4].

Previous studies have found that educational counselors encountering the CSA of adolescent Arab girls revealed feelings of stress, guilt, and personal threat [32]. Likewise, Arab teachers have described coping with CSA as a complex conflict, as they are bound to both professional and cultural community codes [38]. The current study aims to contribute to the understanding of the experiences of kindergarten teachers in contending with the CSA of their students, with a focus on Muslim and Druze Arab kindergarten teachers, who represent religious, traditional communities [37]. The main research question that guided the study was the following: How do Druze and Muslim Arab kindergarten teachers experience coping with CSA cases in their kindergarten?

## 2. Methods

### 2.1. Participants

Fourteen Arab kindergarten teachers (8 Druze and 6 Muslims), all female, were interviewed as part of a broader research project that aimed to describe and analyze coping with CSA among various educational professionals from diverse socio-cultural groups. The participants were selected via criterion sampling based on pre-identified factors [39]. Thus, the researchers initially contacted kindergarten teachers in their immediate area based on the criteria of being exposed to a case or cases of CSA conducted by an adult (e.g., CSA by parents, authority figure, educational employee, or stranger), based on self-identifying themselves as having managed such instances (no formal definition for CSA was provided). Four of the teachers had worked as kindergarten teachers for ten years or less. After locating an initial number of participants, the researchers continued recruitment via the snowball technique [39] and were referred to additional potential interviewees by the participants. The process involved first obtaining the preliminary consent of those potential participants by the informants before their contact information was shared with the researchers.

### 2.2. Data Collection

This study was undertaken by a multidisciplinary team engaged in CSA research. Data collection began with two open interviews conducted by two researchers and one graduate student in education. Based on the emerging themes from the interviews, the team then devised a culturally informed interview guide [40], which was used to conduct 12 additional semi-structured interviews.

The interviews lasted 45–75 min and took place between the years 2019 and 2021. The interview guide included questions about the kindergarten teachers’ knowledge and experience related to incidents or suspicions of CSA in their kindergartens (e.g., Have you ever encountered an incident of CSA?); conduct and implemented practices in incidents of CSA (e.g., Describe your relationships with the child as soon as there is a suspicion of CSA), modes of conduct with parents and in the community (e.g., How do you experience the relationships with the child’s parents after your report? How does your community relate to incidents of CSA of kindergarten students?), relevant professional training and support (e.g., As a kindergarten teacher, have you received any relevant training concerning CSA?), and the effects of encountering a CSA incident on the kindergarten teacher’s personal life (e.g., How does encountering an incident of CSA in your kindergarten affect your personal life?). The interviews were recorded and transcribed. Selected quotes were translated from Arabic and Hebrew into English and presented in each theme. To confirm the translations, randomly selected quotes were back-translated from English into Hebrew or Arabic.

### 2.3. Data Analysis

A qualitative approach was chosen [41], allowing for a multifaceted investigation of kindergarten teachers’ experiences. The data analysis aimed to isolate and articulate the defining elements of the participants’ perceptions [42]. An inductive approach was used to ensure the analysis was not shaped or influenced by prior knowledge or coding frames [43]. To achieve this, the researchers conducted a multi-stage process to synthesize the overall themes and develop a conceptual model that reflected the meanings the kindergarten teachers attached to their experiences with CSA in their classrooms [44]. This began with the researchers thoroughly acquainting themselves with the interview transcripts, entering each into Dedoose software v.9.0.107 (2023). Subsequently, they identified key units of meaning [42], which they sorted into codes and code groups, forming broad themes; when no new issues arose, saturation was achieved. The researchers then revisited the transcripts to further develop the themes that were determined to be central to understanding the essence of the participants’ experiences [45].

### 2.4. Rigor and Trustworthiness

To attain a high standard of rigor and trustworthiness to establish the integrity of the findings [46], the researchers implemented the following strategies: (1) investigator triangulation during some stages of the data analysis, involving a graduate student in education and three experienced researchers; (2) regular peer debriefing regarding the interview transcripts; (3) team debriefing, reflection meetings, and discussions with peers and mentors in the field regarding any preconceptions that could potentially influence the analysis; (4) note-taking and a field diary to encourage reflective awareness; (5) utilizing open interviews and peer consultations to build a guide, which was used for the subsequent interviews, to avoid the data collection being shaped by the researchers’ prior experiences; and (6) member checking by inviting the participants to further corroborate and elaborate on the responses they provided during the data collection [47]. Further member checking regarding cultural and religious nuances was conducted by the Druze and Muslim Arab team members. (7) The data and interpretations were discussed during the weekly peer debriefing sessions to synthesize the units of meaning, confirm that the coding system was organized, consistent, and accurate, and assess the final themes for cohesiveness [48]. (8) Finally, the researchers conferred with experts on CSA, culture, and qualitative research regarding the research process and analysis [49].

### 2.5. Ethical Considerations

This study received approval from the ethics committees of the authors’ affiliated institutions, which also provided the ethical guidelines for this study. Confidentiality and privacy were assured throughout all stages of this study, including transcribing interviews without identifying details, which were kept in a password-protected electronic file. Furthermore, the researchers viewed consent as an ongoing process rather than a one-time event; thus, in addition to obtaining signed informed consent, the researchers repeatedly expressed, verbally and in writing throughout the interview process, that participants were welcome to not answer a particular question or stop the interview entirely at any time [50]. Finally, the researchers conveyed to the participants that if they encountered any emotional difficulty during or after the interview, they could contact the researchers, who would put them in touch with the appropriate professionals. After completing each interview, the researchers also provided contact information directly to the participants for such resources.

## 3. Findings

The analysis yielded a description of the Druze and Muslim Arab kindergarten teachers’ experiences ranging from the extremes of growing fear to proactive coping when contending with CSA cases. The kindergarten teachers encountered children who had experienced a wide range of CSA conducted by an adult. This includes interfamilial CSA (e.g., CSA by siblings, by parents or uncles), as well as CSA in the community (e.g., older youth or not familiarly related adults from the community).

Three main themes emerged. The first theme focuses on the fear that emerged when CSA was revealed. There were two aspects to this fear: the growing awareness of the threat of abuse to their own children and the threats to the teacher’s status and well-being in the community. The second and third themes describe the perceptions and practices implemented due to that fear, both protective and proactive actions. Namely, the second theme demonstrates the protective practices implemented by Druze and Muslim kindergarten teachers to overcome their fear and promote safety for their own children and themselves and as part of their communities. The third theme relates to the teachers’ proactive behaviors in their communities regarding their professionalism and religious perceptions, which set the basis for guiding parents and advocating for the survivors.

### 3.1. “I Began to Be Afraid”: Fear for My Children and Fear for Myself

This theme presents the intersection between the professional role of a kindergarten teacher, being a community member, and being a mother. The teachers’ awareness of threats increased when CSA incidents in their communities were revealed. This fear invaded their lives and their homes. As mothers, they were afraid for their personal children’s safety. Neima, a 48-year-old Muslim kindergarten teacher with 23 years of experience, described the growing awareness of CSA among Arab mothers: “Nearly every day or every week, we hear about such incidents. Due to the increase [in events of CSA], I sense that many mothers have become more alert and worried for their children, especially the younger ones.” The growing awareness of the possibility of CSA invaded their family circle and impacted them where they were most vulnerable—the safety of their own children. Their exposure to CSA incidents in their kindergarten made them feel as though no one was safe. Rania, a 33-year-old Druze kindergarten teacher with 7 years of experience and who is a mother to two girls, shared her thoughts:

I started fearing for my girls. I am afraid to send them to school for fear someone will touch them. I start to be afraid that on their way back from school, perhaps someone will get near them […] This thing enters your life when you see it, and you start imagining how it could happen in your home as well with your children.

The kindergarten teachers were not only afraid for their own children. Due to their involvement in the intervention processes of CSA incidents in their kindergarten, they were subjected to personal threats and violent behavior from community members. Gihan, a 43-year-old Muslim kindergarten teacher with 20 years of experience, described what she went through after reporting a CSA incident in her kindergarten:

The entire neighborhood turned against me. They blamed me instead of taking care of the girl. The person who did it became almost a hero, and I was the person who threatened him. I was helpless, and I didn’t know what to do. I was truly scared. I wasn’t professional enough to speak directly. I should have threatened them instead of them threatening me. I was really frightened.

The sense of fear was evident among the Druze kindergarten teachers as well. Amira, a 55-year-old Druze kindergarten teacher with 30 years of experience and a mother of two, shared an incident that happened to her colleague, who was subjected to intimidation:

You will become an enemy. They send you death threats, like they did to my colleague. They arrived at her house and threatened her and burned her car. [They said] “You disgraced us. Why did you report us to the welfare agency?”

Thus, fear was evident and eminent among both the Druze and Muslim kindergarten teachers. Their professional role in intervening in CSA cases caused fear for their children’s safety, as well as their own. It affected their roles as mothers and as members of their communities.

### 3.2. “I Am More Alert toward My Children”: Implementing Protective Practices in the Family

The kindergarten teachers shared how the overpowering fear invaded their family circle. The hypervigilance brought on by exposure to the CSA of their students invaded their family circle and made them act differently. It pushed them, as mothers, to exhibit more protective practices than in the past. The Druze and Muslim kindergarten teachers mentioned several practices aimed at promoting their children’s safety. The first noted coping mechanism was to observe their children’s conduct and whereabouts and watch them closely. Sania, a 34-year-old Druze kindergarten teacher with 10 years of experience and a mother of two, shared how she acts now, after encountering the CSA of her student:

I am more alert regarding my children. Who are they playing with? Who do they meet? If my daughter goes out, I let her, but I take her and bring her back. If she stays out late, I will wait for her until she gets back.

Similarly, Ahlam, who was taking a break from teaching at the time of the interview to raise her children, described how she constantly speaks to her son: “He tells me everything that happened in kindergarten. I ask him every day what happened. I ask about his teacher, ‘What does she say?’”

The thought that anyone could be a victim and anyone could be a perpetrator affected their interactions with the people around them. “I don’t trust anyone,” Yasmine, a young Muslim kindergarten teacher who was a substitute kindergarten teacher for 3 months, revealed: “I am so afraid for my kids. Even if I send them to their uncles, I pick them up after half an hour. I keep asking them how it was. ‘What did you play? What did you talk about?’” Thus, according to the Druze and Muslim kindergarten teachers, their growing awareness of the threat of CSA caused them to increase their protective behaviors toward their children. They wanted to know everything that happened to their children in the hope that they could identify warning signs sooner and better protect them.

The second approach to coping was teaching their children conduct guidelines to help keep them safe. Rima, a 46-year-old Druze kindergarten teacher with 16 years of experience and a mother of three, elaborated on her approach with her sons:

As for my younger son, I emphasize the need to protect his body by educating him that his body is his and only his. I let him have his privacy in the bathroom. Only I or his father enter. With my older son, I speak seriously that if someone offends you, be sure that he [the offender] is wrong, and he [the offender] is to blame. You must come and tell me.

Rima wanted to encourage her son to share offensive incidents with her, should they occur. She sought to reassure her son and teach him to differentiate between himself, who deserves protection, and the offender, who is always in the wrong and the one to blame. Furthermore, Neima, a Muslim kindergarten teacher and a mother to six children, echoed Rima’s approach, emphasizing

As a mother, I need to help my son build his self-confidence. I need to guide him so he will know that his body belongs to him. No one is allowed to come near him. I need to teach him to be open with me in case he gets hurt.

Both Neima and Rima implemented similar teaching and guiding practices as kindergarten teachers and mothers. The lines between their professional and family circles became blurred as their professional protective practices in the kindergarten were applied in the family circle. Thus, the current theme described how the Druze and Muslim kindergarten teachers reacted to a growing sense of fear originating in the CSA incidents of their students, followed by their professional protective behaviors of elevating protective behaviors toward their children.

### 3.3. “We Need to Raise Awareness and Never Give Up”: Proactive Behaviors within the Professional and Religious Contexts

Two frameworks assisted the Druze and Muslim kindergarten teachers in proactive responses to CSA incidents in their kindergartens. The first—a professional persona—was shared by kindergarten teachers among both Druze and Muslim teachers alike. The second—religious context and beliefs—was addressed differently by the Druze and Muslim teachers.

#### 3.3.1. Professional Persona

Both the Druze and Muslim kindergarten teachers exerted their professional persona to try to overcome the challenges raised by CSA among their students. “I sit with myself, and I think what to do”, explained Yara, a 52-year-old Druze kindergarten teacher with 28 years of experience, who is currently a counselor in kindergartens: “Sometimes I write the stages I need to follow. I need to think about what is allowed and what is forbidden”. The past cannot be altered or deleted, but the future can be planned. Thus, the kindergarten teachers took the time to calculate their steps professionally.

Moreover, they considered it their role to step out of the kindergarten’s borders and to intervene and lead CSA intervention processes for the parents. As professional educators, they were willing to lead the way to make the community safer for the children. Maryam believed parents must also be educated regarding CSA: “You can do many things with the parents. We need to raise awareness and never give up. In case the activities will not be accepted by the parents, we should keep on working and never give up.” Neima further explained: “We can prepare an educational program with the help of the counselor or the psychologist. We should conduct workshops with the parents about signs or behavior that can reveal sexual abuse among the children”. Sania conducted guidance sessions with parents. She explained that they aimed to “Raise awareness of how you should behave with your children, respect their feelings, their privacy. You need to watch children at these ages all the time. You need to be in touch with them, to talk to them”.

Another professional principle held by the kindergarten teachers was to stand with the CSA survivors, regardless of the personal price they might pay. Rania shared the hardships girls endure following CSA, as well as the role she decided to play in the victimized girls’ lives:

The people in the village will scrutinize her and speak badly about her, which can affect her future in the community. I am sure it is not the girl’s fault, and it is not easy for her. I would definitely help her and be by her side no matter how hard it would be for me. I will be on her side.

Thus, the participating kindergarten teachers based their proactive conduct on their professional principles. However, they also incorporated religious values in ways that helped them protect and represent the survivors and guide the parents regarding more appropriate behaviors so CSA incidents could be reduced.

#### 3.3.2. Religious Context and Beliefs

While all of the kindergarten teachers referred to their religious context and beliefs, differences arose based on their religious affiliation. Both the Druze and Muslim kindergarten teachers described their communities as conservative. They acknowledged the religious and social constraints that challenged their intervention processes when encountering CSA incidents involving their students. Maryam, a 40-year-old Muslim kindergarten teacher with 19 years of experience, explained the severity attributed to CSA by the community:

We are a closed, conservative society. We look at the incident of abuse as shameful, a bad thing. We shouldn’t speak about it or discuss it. It is easier to hear about a killing incident rather than about incidents of sexual abuse.

Regarding the reporting of CSA of kindergarten students, there were also barriers. As Neima explained: “They may speak about it, but they will not report it. It is not only that the victim’s family fears for her reputation. They also wish to keep good relationships with their relatives and neighbors”. A similar perception was shared by Rima, who said: “People think too much about what the other family might say, or what the village will say or think”.

However, the majority of the Muslim kindergarten teachers emphasized the challenges religion posed to their intervention in cases of CSA of their students. These challenges were evident throughout the process—from the disclosure, to reporting, to intervention. Ahlam elaborated: “We are a conservative and traditional society, which does not allow us to be free. We don’t discuss sex. It is a non-issue. I never heard of an intervention used by religious people”. Maryam also shared Ahlam’s statements: “Our tradition and ways of life determine what is right and what is wrong. Reporting in cases of CSA is forbidden”.

Conversely, two of the Muslim kindergarten teachers added another perspective on the impact of religion when encountering CSA among young children. They considered religious values as preventing offensive behaviors. Khadija, a 34-year-old Muslim kindergarten teacher with less than 5 years of experience, felt that “People who act this way are not true believers. If we follow our religion, we will not do these things”. Iman, a 43-year-old Muslim kindergarten teacher with 20 years of experience, described how she relied on religious values when addressing the subject in her kindergarten:

I teach the children what is right and what is forbidden based on our religion. They understand. Their parents are religious, too. I tell them we should respect God and be grateful to Him. How? By doing the right thing. God sees us. If we do something wrong, He will sting [punish] us.

The Druze kindergarten teachers shared similar feelings of constraint regarding the disclosure and intervention processes. Like many of the Muslim kindergarten teachers, they referred to their community as a conservative community that does not speak openly about sex or CSA. As Ranya said: “We Druze don’t speak about it. Parents don’t speak about it. We conceal it. We teach it superficially. We won’t go into details”. However, while a minority of the Muslim kindergarten teachers spoke in favor of their religious values in contending with CSA, the majority of the Druze kindergarten teachers felt that their religious beliefs served not only as a prevention tool but also as supportive of the intervention processes. Five of the eight Druze participants considered their religious faith and beliefs as valuable to their intervention processes—both in helping the survivors and in CSA prevention. Yara shared her active usage of religious values when encountering communal resentment in their intervention processes:

Our religion and values can help us. I always incorporate our values and our religion when I speak to the parents. […] Religion helps me to speak to parents in a more positive manner. Based on our Druze values, we should not forsake her [the survivor] […] We are like brothers to one another.

Amira reasserted Yara’s statements. She did not see the Druze religion as an obstacle. On the contrary, she stated that “If people would follow religious laws, there wouldn’t be sexual abuse. […] It can be an assistance to stop the cycle of abuse”. Her words echoed those of Khadija, as both felt that true believers do not offend one another. Thus, the road to a life without CSA is based on religious faith.

The internal processes of the Druze and Muslim kindergarten teachers showed that fear invaded their family, professional, and community circles and drove them to implement proactive behaviors. This resulted in a system of increased protection in their family circle and proactive professional intervention processes in the community circle. Differences among the Druze and Muslim kindergarten teachers were apparent regarding the role religion might play in their intervention practices.

## 4. Discussion

CSA in early childhood can be devastating not only for the survivors but also for their communities, including their teachers [27]. The current study revealed the startling impact CSA had on kindergarten teachers’ lives as fear invaded the three circles of their kindergarten, families, and communities. However, the origins of fear for each circle were distinct. The disclosure of CSA in their kindergarten exposed the participating kindergarten teachers to ‘toxic knowledge,’ which consequently altered their perceptions and conduct [27].

The perception that danger lies everywhere intensified their sense of fear for the safety of their own children. In the community circle, fear was aroused due to resentment and anger from the parents and community members regarding the revelation of the ‘secret’ and endangering family honor. These reactions have been found to be pervasive among closed conservative societies, such as Muslim and Druze communities [30,32].

The need to overprotect their children in light of the possible danger drove the participating kindergarten teachers to implement proactive parenting. The feeling that no one is safe anywhere [51] urged them to constantly watch, escort, investigate, guide, and worry about their children. Their proactive conduct in their communities occurred on two levels: (1) their professional identity and (2) their religious context and beliefs. Kindergarten teachers are defined and perceived as educational and organizational leaders of their kindergarten [7]. Their professional identity guides them to protect CSA survivors and lead the intervention process as part of their role as kindergarten teachers. As educational and organizational leaders, their actions are reflected in their perceived role, namely the responsibility for the well-being of their students. This perception of a kindergarten teacher’s role is supported by earlier research (e.g., [9,52]).

However, intervention processes in conservative closed communities can be challenging and complicated due to possible negative reactions from the close surroundings. Both the Druze and Muslim kindergarten teachers confirmed the complexity described in previous research [29,53]. When sharing their personal perceptions of religious values on intervention processes in cases of CSA, the Muslim kindergarten teachers emphasized the obstacles their religious community poses to CSA prevention and intervention processes among their students (e.g., [30,32]). However, there were some who considered religious values to be supportive of intervention processes in CSA cases. These voices were more evident among the Druze kindergarten teachers who considered their religion to be a positive source to fight CSA in their communities. It could be postulated that the greater integration of the Druze community into Israeli secular society resulted in a more open approach to CSA intervention.

The Druze and Muslim kindergarten teachers were aware of possible objections to their interventions [30,32] and adapted their interventions accordingly. Rather than rejecting their traditional and communal values, they incorporated them into their dialogue with the parents. Their choice to implement culturally sensitive intervention processes has been recommended in several studies (e.g., [6,37,54,55]). Based on the current study’s findings, it appeared that the participants from the Druze community identified more with this strategy of implementing culturally based interventions than the Muslim participants.

The kindergarten teachers designed and implemented professional sessions to guide parents both as part of their routine work and in times of crisis when CSA was exposed. Their proactive guidance for parents relied on their professional knowledge, strengthened by other professional personnel, such as kindergarten counselors and psychologists. Their actions emphasized their role as educational and organizational leaders, embedded in their role descriptions [4,8,52]. Their persistence in maintaining a professional intervention process reflected the findings in [56] regarding multiple professionals implementing a cross-disciplinary intervention in cases of child abuse.

Furthermore, the Druze and Muslim kindergarten teachers were aware of the complexity of these intervention processes. They experienced a conflict between the cultural codes of their communities and their obligations as professional kindergarten teachers to help their students. These feelings have been previously described as entrapment [38]. To overcome their sense of entrapment, the kindergarten teachers took control of the intervention process, knowing it needed their professional management and supervision [56]. Their choice to be proactive, despite their fear and the threats they faced, exposed them to an inner conflict between their personal and professional values and their religious and community values. This conflict has also been reported in a study of Arab school counselors who worked with female adolescents who were sexually abused [32]. Their cultural and personal backgrounds could have led them to maintain the traditional conservative community and family reactions to CSA, such as keeping it a secret [37]. Nonetheless, rather than submitting to fears and threats, they bravely chose to rise to the challenge, recruiting both their professional identity and religious beliefs as anchors.

In summary, an encounter with CSA of kindergarten students elicited fear in the three circles in which kindergarten teachers operate—the kindergarten, the family, and the community. Their fear originated from their exposure to ‘toxic knowledge’ [27] and critical and offensive responses in their communities. In the face of this fear, the kindergarten teachers chose to implement proactive actions, resulting in the overprotection of their students and their own children. Another line of action was offering professional guidance to parents. Many participants, mainly from the Druze community, referred to incorporating religious values as part of their professional intervention and guidance processes.

### 4.1. Limitations

Firstly, as this is a qualitative study, the findings sought to provide an in-depth exploration of the phenomenon of how kindergarten teachers experience coping with CSA. However, the structure of kindergartens in the Israeli context may not represent other professionals’ experiences. Consequently, this study may be limited in how its findings can be generalized [57]. In addition, the sample size was small. Furthermore, the sample was restricted to Muslim and Druze Arab kindergarten teachers, two communities with similar characteristics—strong religious foundations and patriarchal characteristics. Although there are key similarities, due to the limited sample size, it was not possible to analyze the socio-religious groups separately. It can be assumed that kindergarten teachers who do not live and work in a religious context will face different challenges. Future research should further investigate the implications of cultural context on professional conduct and values.

Furthermore, while the current study highlights the often-unheard perspectives of Arab kindergarten teachers in Israel, it does not delve into the participants’ cultural construction of CSA. Exploring this topic should be an essential focus for future research. Moreover, this study exclusively examined CSA cases perpetrated by adults, reflecting the incidents identified within the sample. Given the literature’s insights on peer victimization and problematic sexual behaviors (PSBs) among kindergarten children (e.g., [58]), and the significant differences in characteristics and definitions of these phenomena compared to adult-perpetrated CSA, it is crucial to explore kindergarteners’ perceptions regarding these prevalent issues further. Finally, the sample contained only female participants, which could bias the findings, especially in light of what is known about the role of gender in reporting awareness [59]. This asymmetry can be explained due to the feminization of the teaching profession especially in early childhood education [60]. Further research should examine the context of gender in managing complex situations, such as child abuse and neglect.

### 4.2. Implications for Theory, Research, and Practice

The current study, while emphasizing the willingness of Druze and Muslim Arab kindergarten teachers to lead and guide intervention processes, revealed personal and community obstacles. The fear that expanded into the family and community circles was alarming, as it had the potential to inhibit the kindergarten teachers’ motivation and abilities to implement CSA interventions. Future research should examine kindergarten teachers’ personal, as opposed to professional, needs in order to plan and offer supportive resources in cases of CSA among kindergarten children. The differences in the findings between the Druze and Muslim kindergarten teachers should be researched further in a larger sample to gain greater rigor and trustworthiness.

Although the kindergarten teachers shared their adapted intervention processes with parents, it is apparent that there is a need for a more formal and structured intervention process that can be implemented in more conservative kindergartens and communities. It is vital that kindergarten teachers are continuously taught and guided regarding appropriate conduct in the event of a CSA incident so that they will not solely carry the burden and will be supported by formal, professional knowledge. Kindergarten teachers are willing to fulfill their educational role as leaders [52] when things are calm as well as in times of stress. It is advised that a comprehensive professional and personal support system that is culturally relevant would be available to all kindergarten teachers, especially when they need to take the leading educational role in an incident of CSA among younger children.

## Data Availability

The data presented in this study are available on request from the corresponding author.
